# Preparing for a “dirty bomb” attack: the optimum mix of medical countermeasure resources

**DOI:** 10.1186/s40779-020-00291-3

**Published:** 2021-01-17

**Authors:** Alexis Rump, Patrick Ostheim, Stefan Eder, Cornelius Hermann, Michael Abend, Matthias Port

**Affiliations:** Bundeswehr Institute of Radiobiology, Neuherberg Str. 11, 80937 Munich, Germany

**Keywords:** Radiological emergency, Dirty bomb, Medical countermeasures, Radionuclide incorporation, Decorporation treatment, Screening, Efficiency, Data envelopment analysis

## Abstract

**Background:**

In radiological emergencies with radionuclide incorporation, decorporation treatment is particularly effective if started early. Treating all people potentially contaminated (“urgent treatment”) may require large antidote stockpiles. An efficacious way to reduce antidote requirements is by using radioactivity screening equipment. We analyzed the suitability of such equipment for triage purposes and determined the most efficient mix of screening units and antidote daily doses.

**Methods:**

The committed effective doses corresponding to activities within the detection limits of monitoring portals and mobile whole-body counters were used to assess their usefulness as triage tools. To determine the optimal resource mix, we departed from a large-scale scenario (60,000 victims) and based on purchase prices of antidotes and screening equipment in Germany, we calculated efficiencies of different combinations of medical countermeasure resources by data envelopment analysis. Cost-effectiveness was expressed as the costs per life year saved and compared to risk reduction opportunities in other sectors of society as well as the values of a statistical life.

**Results:**

Monitoring portals are adequate instruments for a sensitive triage after cesium-137 exposure with a high screening throughput. For the detection of americium-241 whole-body counters with a lower daily screening capacity per unit are needed. Assuming that 1% of the potentially contaminated patients actually need decorporation treatment, an efficient resource mix includes 6 monitoring portals and 25 mobile whole-body counters. The optimum mix depends on price discounts and in particular the fraction of victims actually needing treatment. The cost-effectiveness of preparedness for a “dirty bomb” attack is less than for common health care, but costs for a life year saved are less than for many risk-reduction interventions in the environmental sector.

**Conclusion:**

To achieve economic efficiency a high daily screening capacity is of major importance to substantially decrease the required amount of antidote doses. Among the determinants of the number of equipment units needed, the fraction of the potentially contaminated victims that actually needs treatment is the most difficult to assess. Judging cost-effectiveness of the preparedness for “dirty bomb” attacks is an issue of principle that must be dealt with by political leaders.

## Background

A radiological dispersal device (RDD, “dirty bomb”) is a conventional explosive combined with radioactive material. Its construction is much simpler than an improvised nuclear device as it requires only basic skills. Radioactive sources are widely used in industry, research and medicine [1, 2] and it seems relatively easy to get access to such material. The find of abandoned radioactive material, although mostly in lower activities, is regularly reported [3]. But “orphan sources” of radioactivity have resulted in large-scale scenarios in several cases in the past as for example in Goiania [4]. The theft or purchase of radioactive sources on the black market represents another possibility to get radioactive materials.

A terrorist attack with a radiological weapon has never happened up to now. However, several incidents suggest that such attacks were planned and on the way to be prepared: In 1995 authorities found a container of radioactive cesium in Moscow’s Ismailovsky Park after a TV station had been contacted about the cache. In 1998 a container filled with radioactive material was found attached to a mine hidden near a railway line in Chechnya. In 2004 a large number of household smoke detectors containing small quantities of americium-241 were found at a raid of a terrorist cell cache in London [5–7]. These few examples show that the attack by a radiological dispersal device (“dirty bomb”) must be considered as a serious terrorist threat [8, 9].

The way how a dirty bomb attack will occur and the means used cannot be predicted and the probability of occurrence cannot be quantified. It seems however reasonable to assume that radionuclide(s) widely used for commercial civilian applications and therefore easily available are of particular concern. High activities in order to cause a maximum of disruption may be of particular interest to terrorists. Cesium-137 is included in industrial irradiators at high activity levels in a powdered salt form (CsCl). Thus in combination with an explosive, it can be easily dispersed, and considering the high activities in the sources the contamination of a large area may be expected (“area denial dirty bomb”) [10]. This is less the case with cobalt-60: Although it is also used at high activities in industrial irradiators or cancer treating teletherapy machines, the radioactive material is in form of small metal pellets. Therefore, aerosolization will be much less [11, 12] and the maximum range of the fragments flying out of the “dirty bomb” are expected to be much more limited causing a scenario of a very different kind compared to cesium-137 [10]. Both radionuclides mentioned emit gamma radiation with a long range in air and can easily be measured with radiation detection devices. This is not the case for nuclides emitting only or mainly alpha radiation, like for example americium-241, also widely available and prone to be misused in a dirty bomb [3].

From a medical point of view, victims of a dirty bomb attack may suffer blast injuries with mechanical trauma due to fragments and burns. These injuries may be life threatening and according to the principle “treat first what kills first” should be treated with first priority [13, 14]. External irradiation may occur, but the analysis of many hypothetical scenarios suggests that the radiological doses absorbed by most victims would not be sufficient to cause acute radiation sickness, at least in the case of an area denial bomb attack with cesium-137. However, deterministic radiation damages cannot be excluded in all cases, and when assessing victims, it is crucial to take into account that even prodromal symptoms may show up only hours after irradiation. Besides mechanical trauma and possible external irradiation, a much larger number of people may be externally contaminated more or less with radioactive material and this is always associated with the danger of radionuclide incorporation and internal contamination [13]. The occurrence of acute radiation sickness is usually not to be expected from radionuclide(s) incorporation [15], except in special cases as the Litvinenko poisoning [16]. However, internal contamination and irradiation may cause stochastic health effects (e.g., cancer) in the long run.

The elimination of radionuclide(s) out of the body can be enhanced by the administration of decorporation agents, and thus the radiological dose absorbed and the negative health impact can be reduced. For nuclear and radiological emergencies, the main antidotes used for decorporation are Ca(diethylenetriaminepentaacetic acid, DTPA) and Prussian Blue [17–19]. Therapeutic efficacy decreases if treatment initiation is delayed after incorporation and in most cases there is a time slot of hours to several days to achieve optimal results, depending on the radionuclide(s), the physicochemical properties of the compound and the invasion pathway [20, 21]. Therefore, it seems prudent from a medical point of view to start treatment already if radionuclide(s) incorporation is only suspected until a substantial intake is excluded by measurement. This “urgent approach” treatment strategy seems particularly justified, as the adverse effect of short-term treatments with Ca(DTPA) or Prussian Blue have been shown to be slight [19, 22]. In the case of a large number of victims who are potentially internally contaminated, as can be expected after a “dirty bomb” attack, this “urgent approach” strategy requires however a large number of antidote daily doses that must be available in stock [23].

An additional factor determining the size of the required stockpile is treatment duration. For the assessment of the needs of an international stockpile, the Radio-Nuclear Working Group of the WHO departed from 10 to 12 days [24]. The manufacturers of the antidotes recommend a treatment duration of at least 30 days for Prussian Blue [25] and an irregular dosing scheme over several weeks for Ca(DTPA), depending lastly on laboratory results [26]. According to our simulations of therapeutic schemes, therapeutic efficacy reaches a plateau after about 90 days of treatment for some (e.g. cesium-137) but not obviously for all radionuclide(s) [20]. Assuming a scenario with 60,000 potentially contaminated patients (e.g. US National Planning Scenario Nr. 11) [27] and treating all victims for 90 days for decorporation would require 5,400,000 antidote daily doses and an immense logistic challenge.

However, probably only a fraction of the potentially contaminated patients, that is a priori unknown, will actually need treatment [23, 24]. Rapidly identifying these patients, or in the case of an “urgent approach” strategy rapidly identifying victims that have not incorporated radioactivity in substantial amounts to rapidly exclude them from further treatment, permits to strongly reduce the number of daily doses of antidotes needed [23]. The triage of patients is not possible by simple clinical examination, but only by whole-body measurement of radioactivity after superficial decontamination. The measurement of radioactivity is possible with mobile equipment, but the requirements on the devices and detection limits depend on the radionuclide(s) because of different types and energies of the emitted radiation [28]. Monitoring portals permit a high measurement throughput with a limited detection limit. Mobile whole-body counters with heavier shielding have a higher sensitivity, but the time needed to screen a single patient is also longer. In the first part of our analysis, we assessed the suitability of this equipment for the screening of victims potentially contaminated with cesium-137 (gamma radiation emitter by its short-lived daughter barium-137 m) or americium-241 (alpha radiation emitter with an additional low energy gamma radiation emittance).

There is trade-off between the number of antidote daily doses needed for preparedness and the screening capacities, i.e. the number of screening equipment units [23]. The goal should be to achieve the best medical treatment efficacy with a minimum of resources, i.e. the lowest possible financial investment. In the second part of the present analysis, we therefore determined the optimum resource mix by calculating efficiencies resulting from different combinations of antidote stockpile sizes and numbers of screening units. Efficiency must be conceptually distinguished from efficacy as it quantitatively relates an input to an output. Therefore a (health) system can have a high efficiency even if its efficacy is highly mediocre. In our study, we analyzed efficiency for a preparedness level achieving the best medical result (“urgent approach” strategy, treatment duration sustained for 90 days if indicated) and we did not consider options leading to less satisfactory medical results, even if financial investments are obviously lower and reveal to have higher efficiency.

## Methods

### Calculation of committed effective doses corresponding to technical detection limits

Radiation exposure can be quantitated by the committed effective dose that is defined as the total effective dose due to radionuclide incorporation absorbed over 50 years after the incident (70 years for children). This dose cannot be directly measured by a sensing device like the dose rate of radiation emanating from a source in the environment, but it requires the measurement of radioactivity in the body, followed by internal dosimetry calculations based on biokinetic-dosimetric models [13]. Different values of the committed effective dose have been proposed as thresholds for the indication of a decorporation treatment (“action levels”) ranging from 20 to 200 mSv [19, 29–31]. Radioactivity detection equipment must permit to measure activities leading to the defined threshold values in order to identify victims actually needing treatment. As after acute intake, the activity in the body will decrease by physical decay, but also in many cases predominantly by biological elimination, the required detection sensitivity will depend on the radionuclide involved as well as the time period necessary to screen the victims. Assuming that a full treatment course lasts 90 days, as at least for certain radionuclides efficacy shows a plateau at that time point after acute exposure, the screening equipment should ideally be able to still detect an amount of radioactivity at *t =* 90 days corresponding to the committed effective dose set as decorporation treatment indication threshold. If opting for the implementation of an “urgent approach” strategy for decorporation, this would also be required in order to fully use the screening capacities to reduce the antidote stockpile requirements according to the algorithm previously described [23].

**Fig. 1:**
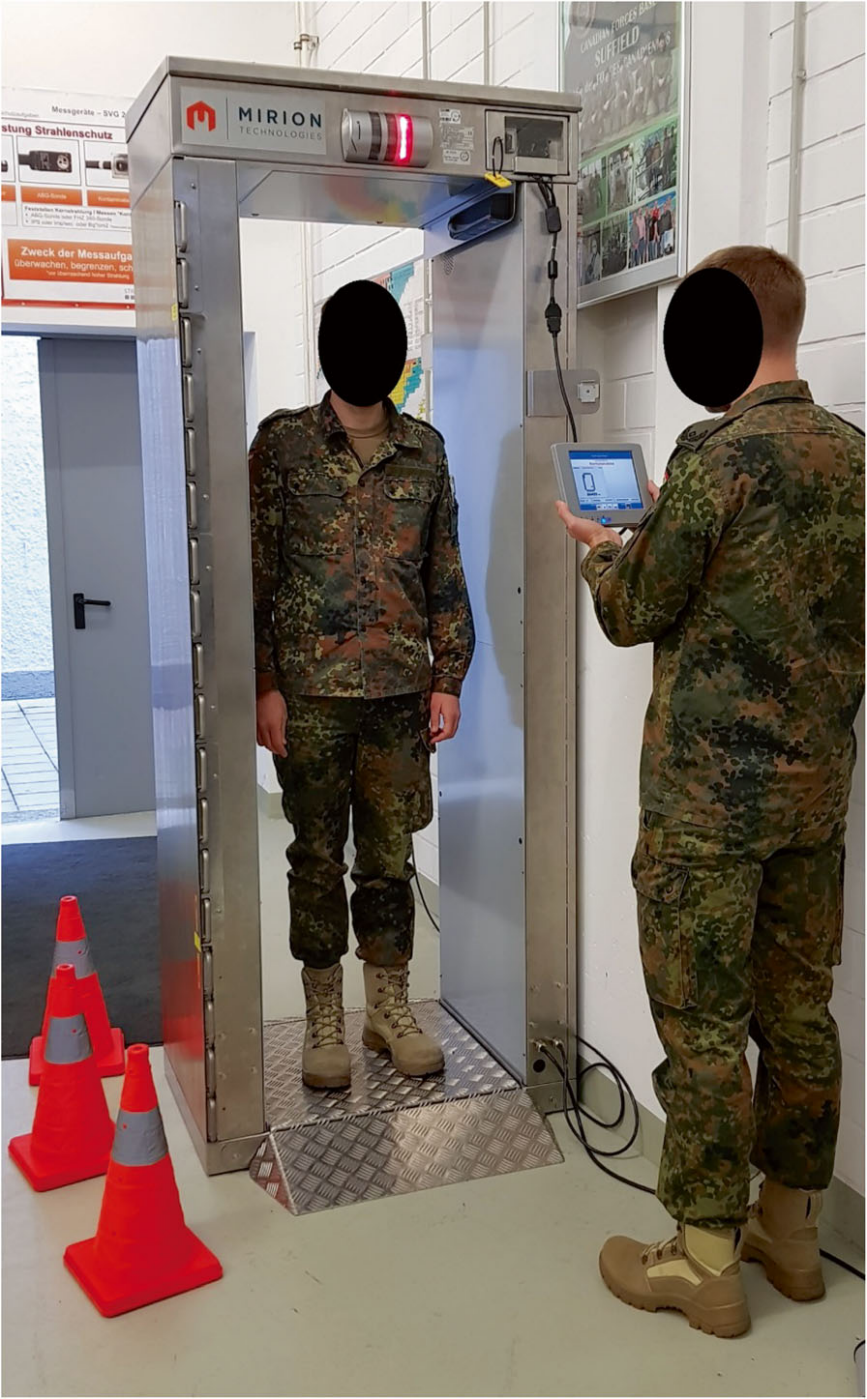
The monitoring portal used at the Bundeswehr Institute of Radiobiology (CheckPoint: Gate™ FastTrack-Fibre™ Mobile) with technical specifications. Detection limits are given for different measurement modes and times [32]. Lead shielding 15 mm: cesium-137 [wait in (5 s): 1.35 kBq; walkthrough (0.57 s): 4.8 kBq], americium-241 [wait in (5 s): 29 kBq; walkthrough (0.57 s): 105 kBq]. Lead shielding 30 mm: cesium-137 [wait in (5 s): 1.25 kBq; walkthrough (0.57 s): 4.5 kBq], americium-241 [wait in (5 s): 28 kBq; walkthrough (0.57 s): 100 kBq]

Based on the technical specifications of a monitoring portal used at the Bundeswehr Institute of Radiobiology (Gate™ FastTrack-Fibre™ Mobile, Mirion Technologies, San Ramon, CA, US) (Fig. 1) [32], we computed the committed effective doses corresponding to activities detectable up to 90 days after acute intake for cesium-137 or americium-241. Both radionuclides were considered, as besides alpha radiation americium-241 emits gamma-radiation of lower energy as cesium-137 (59.5 keV vs. 662 keV for cesium-137) and monitoring portals are known to have a limited sensitivity particularly at gamma energies below 200 keV [31]. The different measurement modes available were taken into account. This permits to assess whether a monitoring portal is a suitable screening equipment to make decisions on the indication of a decorporation treatment or whether more sensitive whole-body counters are needed. All computations were performed using the commercial Integrated Modules for Bioassay Analysis (IMBA, National Radiological Protection Board, United Kingdom) software [33].

### Calculation of the efficiency and cost-effectiveness of various medical countermeasure resource mixes

#### Scenario and basic assumptions on radionuclide incorporation

We assumed a large-scale scenario of a dirty bomb attack containing cesium-137 as described by the federal interagency community in the US National Planning Scenarios (Nr. 11) [27] in order to identify the “range of response requirements” and to permit a capabilities-based planning process: At each of 3 sites where bombs detonated almost simultaneously there are 180 fatalities, 270 injured people requiring medical care and up to 20,000 people having superficial radioactive contamination, i.e. a total of 60,000 people (3 × 20,000 = 60,000) potentially also internally contaminated. There are no victims that will develop an acute radiation syndrome in the further course.

In our simulations, we assumed inhalations of a soluble cesium compound (type “F”) or (differing from the original scenario) a medium to poorly soluble americium compound (type “M”), both leading to similar committed effective doses of 200 mSv, as this dose is usually considered as a clear indication for decorporation treatment without being expected to cause deterministic radiation damage.

#### Antidote requirements and assumptions on screening capacities

**Fig. 2:**
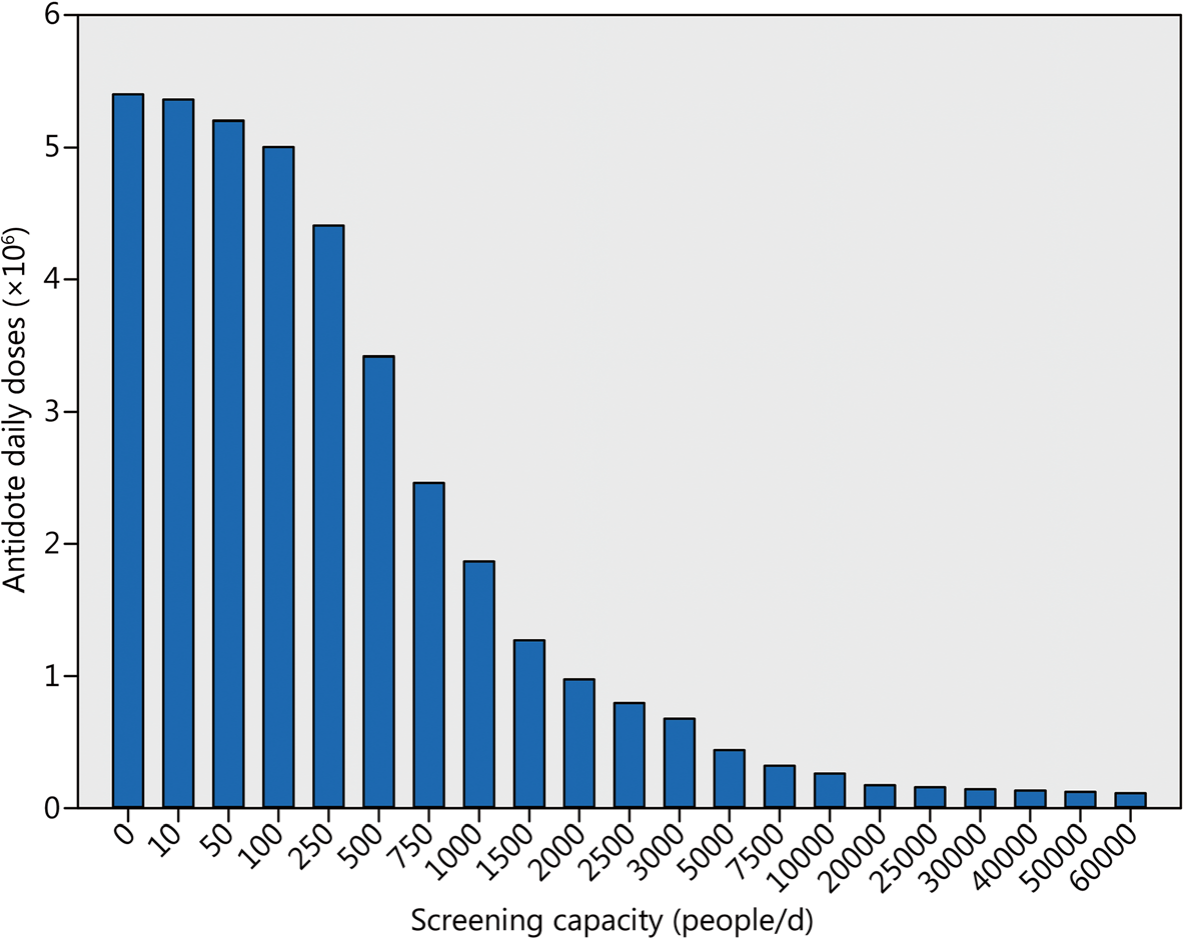
Number of antidote daily doses needed for an “urgent approach” treatment strategy and a large-scale scenario with 60,000 potentially internally contaminated victims depending on the screening capacities (monitoring portals or whole-body counters). Assumption: 1% of the potentially contaminated victims actually need treatment. Treatment duration 90 days. Figures are independent on the radionuclide(s) involved. Computations according to the algorithm described in [23]

For decorporation of cesium-137, the usually recommended dosage of Prussian Blue (Radiogardase®) is 3 × 1 g/d per os, and in the case of americium-241, the administration of Ca(DTPA) 0.5–1 g/d intravenously is indicated [18, 19, 34]. The total antidote daily doses needed to implement an “urgent strategy” treatment approach depending on the screening capacities permitting to identify victims actually needing or not needing continuation of treatment were calculated according to an algorithm previously published [23]. For a 90-day treatment, antidote requirements for the implementation of an “urgent approach” strategy and different screening capacities are shown in Fig. 2.

Taking into account measurement modes and times as well as organizational frictions, we considered that using a monitoring portal, screening 15 persons/min (total time per person: 4 s) is possible for a technically trained team. This corresponds to roughly 1000 persons/h and assuming an effective working day of 10 h (without breaks) 10,000 victims/d. An effective 10 h working day has been assumed as it is a figure found in some official documents on nuclear emergency preparedness [35].

Detecting an activity of 250 Bq americium-241 by a 10 min measurement in a mass casualty situation does not seem absolutely necessary to us for triage purposes in a radiological emergency. That’s why in the case of a measurement in a mobile whole-body counter, taking into account the variability of the detection limit in relation to the corresponding radiological doses as well as practical constraints (installation of the patient, short explanations), we assumed an hourly capacity of roughly 10 people/h (i.e. 6 min and not 10 min for the whole procedure) and again an effective working day of 10 h. Thus, we departed from the assumption that 100 people/d may be examined per whole-body counter unit. A capacity of 10 people/h is a value that has also been given for entire body scans in the TMT Handbook [31].

The possibility to reduce measurement times seems limited for practical reasons or detection limits. However, extending the effective working time per day seems a more realistic option, provided to ascertain that an adequate number of technically trained staff is available. We therefore also considered the impact on efficiency and the costs incurred for the gain of a statistical life year if extending the daily working time from 10 to 20 h, i.e. doubling the daily screening capacity with the same number of screening units.

#### Evaluation of the lifetime saved

The committed effective doses and efficacy of treatment were assessed based on the ICRP models for cesium-137 and americium-241 as described previously [20]. We assumed inhalation of cesium-137 or americium-241 activities leading to similar committed effective doses of 200 mSv. We also assumed decorporation treatment to be started 12 h after acute radionuclide exposure in all victims until a relevant incorporation activity has been excluded by measurement. Treatment efficacy was expressed as the complementary value of the dose reduction factor:

$$ \mathrm{Efficacy}=1-\left(\mathrm{dose}\ \mathrm{with}\ \mathrm{treatment}/\mathrm{dose}\ \mathrm{with}\mathrm{out}\ \mathrm{treatment}\right) $$

Daily treatment was sustained for 90 days in victims with confirmed indication, leading to a therapeutic efficacy of 53.8% after cesium-137 incorporation and 54.2% for americium-241 [20]. The absorption of an effective dose of 1 mSv has been reported to reduce statistical lifetime expectation by 0.42 days, averaged over both genders and all age groups [36]. Thus, the statistical lifetime saved by decorporation treatment was calculated by multiplication of the radiological dose reduction in mSv with 0.42 d/mSv. As the reduction of statistical lifetime caused by the absorption of 1 mSv effective dose depends on age [36], we repeated our computations for different age groups.

Depending on the scenario, the fraction of the potentially contaminated victims actually needing treatment will vary and is a priori unknown. Quite divergent figures have been suggested: 1% according to the Radio-Nuclear Working Group of the WHO [24]; 40–60% according to the US Department of Health & Human Services [37]. That’s why in our calculations, we varied the percentage of victims actually needing decorporation treatment from 0.01 to 100%. Moreover, as a further factor determining the statistical lifetime saved, we analyzed the impact of the threshold level fixed to decide on the indication for a decorporation treatment between 20 mSv up to 500 mSv (this latter value is far above the usual level of 200 mSv considered as clear indication threshold, but has been chosen for sensitivity analysis to visualize the impact of the threshold level).

#### Evaluation of costs

Costs of antidotes were based on purchase prices at pharmacies in Germany. The purchase price of 1 package Radiogardase® with 36 capsules a 500 mg Prussian Blue each was determined with 101.38 € and assuming a usual dosage of 3 g (6 capsules) /d with 16.90 €/d. The price of 1 package of Ca(DTPA)-Heyl® with 5 ampules à 1 g amounts to 72.95 € in Germany and thus a treatment with 1 ampulla/d costs 14.59 €/d. To account for the fact that authorities or hospitals may directly purchase from the manufacturers and that rebates have to be expected, we also considered the costs if purchasing with 30, 50% or 70% discounts relative to pharmacy prices. The costs related to acquisition transactions as well as of the holding phase including the renewal of the stocks because of the limited shelf-life of medication were not taken into account.

For a monitoring portal with the specifications required, we estimated the purchase costs with 100,000 €. We assumed higher purchase costs of 500,000 € for a mobile whole-body counter. As the precise costs will strongly depend on specific requirements and the number of units ordered, we performed a sensitivity analysis and varied the prices in a range between − 50 and + 50% of our assumptions.

#### Computation of efficiency by data envelopment analysis

Data envelopment analysis is a methodology to assess the relative efficiency of a set of decision-making units [38]. It does not depart from a defined production function as stochastic frontier analysis, but is a non-parametric method and can be used even on a small sample size. It has been widely used in health economics for efficiency assessment at levels ranging from single hospitals (microeconomic level) to health systems as a whole (macroeconomic level) [39–41]. A further advantage is the possible inclusion of multiple inputs and outputs valued either in natural/physical or monetary units.

In our case, the screening equipment and the antidotes represent the input. As the prices of both are known (antidotes) or can be reasonably assessed (equipment), the whole costs that have to be incurred can be expressed as a single input in a monetary value. In data envelopment analysis the best efficiency value for each decision making unit is computed by calculating weights for multiple inputs and outputs and optimal efficiency is often associated with a weight of zero for individual items for which a unit performs less well. This also means that efficiency corresponds to the concept of Farrel and not Pareto-Koopman [42]. This issue is avoided as in our case there is only a single input (total costs) and output (statistical lifetime saved), and efficiency is just computed as the ratio of productivity (statistical lifetime saved/total costs) relative to the highest productivity value for the scenario considered including all assumptions. For a given resource mix option i, this means put into a formula:

$$ \mathrm{Efficiency}\ \mathrm{i}=\left(\mathrm{Productivity}\right)\ \mathrm{i}/\left(\mathrm{Productivity}\right)\max =\left(\mathrm{lifetime}\ \mathrm{saved}/\mathrm{costs}\right)\ \mathrm{i}/\left(\mathrm{lifetime}\ \mathrm{saved}/\mathrm{costs}\right)\ \max $$

At the same time, the inverse of the highest productivity represents the costs of a statistical life year saved and therefore permits an assessment as in cost-effectiveness analysis.

In our data envelopment analysis, we used an input orientation as we consider that the output is set by the scenario and the highest medical benefit for the patients should be sought by implementing an “urgent treatment” strategy. Thus, only the investments in preparedness can be controlled. As accepting a reduction of the best achievable gain of lifetime is not an option for us, we considered only efficiency scores using a constant (CRS) and not a variable return on scale (VRS).

Our simulations are based on a binary distribution (no treatment or treatment), but we assume that all treated victims would absorb exactly the committed effective dose fixed as an indication threshold (and no higher dose) if remaining untreated. In a real setting, this is not realistic and there will be a more or less, but unpredictable variability in the committed effective doses absorbed by victims exceeding the dose threshold fixed. This means that the individual and average lifetime saved per person, as well as the total lifetime saved for all victims in reality will exceed our computed values. As long as the distribution of the committed effective doses remains constant in the victim population, this will however not affect efficiency values. But the costs to save a statistical life year will vary depending on the concrete distribution and the values of our computations are the highest costs for the dose threshold fixed (as the total lifetime saved is at a minimum). Our calculations for the costs of a saved statistical life year therefore lead to very conservative estimations.

## Results

### Technical detection limits and the suitability of monitoring portals and mobile body counters for triage purposes

In order to fully use the screening capacity to reduce antidote stockpiling requirements, the detection limit of the screening equipment should permit the identification of victims actually needing decorporation treatment up to 90 days after the incident.

A body burden of 4.8 kBq (detection limit of the monitoring portal) of cesium-137 at the time of acute radioactivity intake corresponds to the inhalation of 5.85 kBq (amount including the deposited + exhaled activity) and leads to a 50 year committed effective dose of 39.2 μSv. In order to be able to stay over the detection level [32] all over the treatment period using a “walkthrough” mode (i.e. body burden 4.8 kBq at *t =* 90 days), the initial body burden at *t =* 0 should exceed 9.4 kBq and the inhaled activity 11.5 kBq. However, in the case of an “urgent treatment” approach, it must be taken into account that Prussian Blue speeds up elimination. Without treatment, the retention of cesium-137 follows a two exponential decay function (R(t) = 0.1 × *e* ^(− 0.347 × *t*)^ + 0.9 × *e*^(− 0.00630 × *t*)^) and the biological half-life is in a range of 70 to 130 days [43]. Although results vary, it was described that Prussian Blue decreases the elimination half-time of cesium-137 by about 65% [18]. We assumed that the half-life was reduced from 110 to 40 days and calculated that a remaining activity of 4.8 kBq after 90 days of treatment would correspond to an initital intake of 25.8 kBq and inhalation of 31.5 kBq. If untreated, this would lead to a committed effective dose of 211 μSv. Using the more sensitive “wait in” mode (detection limit 1.35 kBq) would require the intake of 7.27 kBq (inhaled activity 8.86 kBq) leading to a dose of 59.4 μSv. Therefore, even if selecting a very strict limit for decorporation treatment indication (e.g. committed effective dose of 20 mSv), a monitoring portal is a suitable screening equipment in the case of cesium-137 incorporation and is a good mean to reduce the antidote amount requirements.

In the case of americium-241 (solubility type “M”, recommended as default type), a body burden of 105 kBq at the time of acute radioactivity intake, corresponding to the detection limit in the “walkthrough” mode, would be achieved by the inhalation of 128 kBq and lead to a committed effective dose of 3470 mSv. Even if using a “wait in” mode and reinforced lead shielding of 30 mm instead of 15 mm, a body burden of 28 kBq (detection limit, corresponding to an inhalation of 34 kBq) at the time immediately after incorporation would still lead to a dose of 922 mSv. In the case of an insoluble compound (type “S”), 28 kBq body burden at *t =* 0 would lead to a dose of still 292 mSv and in the case of a soluble compound (type “F”) to 3810 mSv. Considering these very high dose values, it does not seem worth to deal with even higher activities permitting to still detect americium-241 after 90 days. Besides detecting very high activities for orientation, monitoring portals do not seem to be the right screening equipment to decide meaningfully on the indication of a decorporation treatment. For that purpose whole-body counters seem to be required [44].

### The impact of screening equipment-antidote combinations on preparedness efficiency and cost-effectiveness

**Fig. 3:**
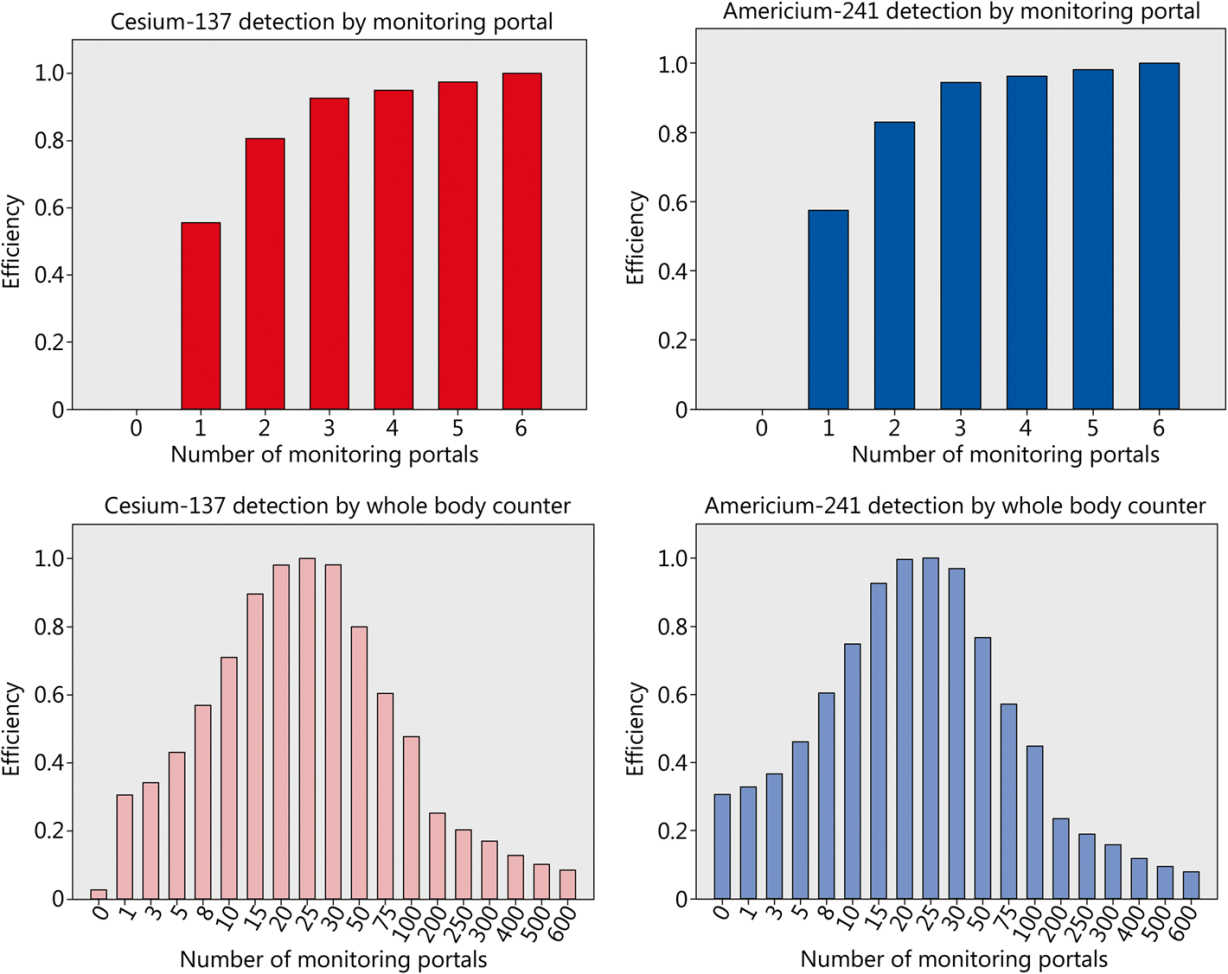
Efficiency of combinations involving various numbers of screening equipment units to detect cesium-137 or americium-241. Decorporation treatment is started according to the “urgent treatment” approach 12 h after acute radioactivity incorporation with Prussian Blue for cesium-137 or Ca(DTPA) for americium-241. Inputs are measured in monetary units based on prices in Germany and statistical life time saved as output. Efficiency values for americium-241 and portal monitors are shown for completeness, but portal monitors are not an acceptable option for that purpose because of their limited technical detection limits

**Fig. 4:**
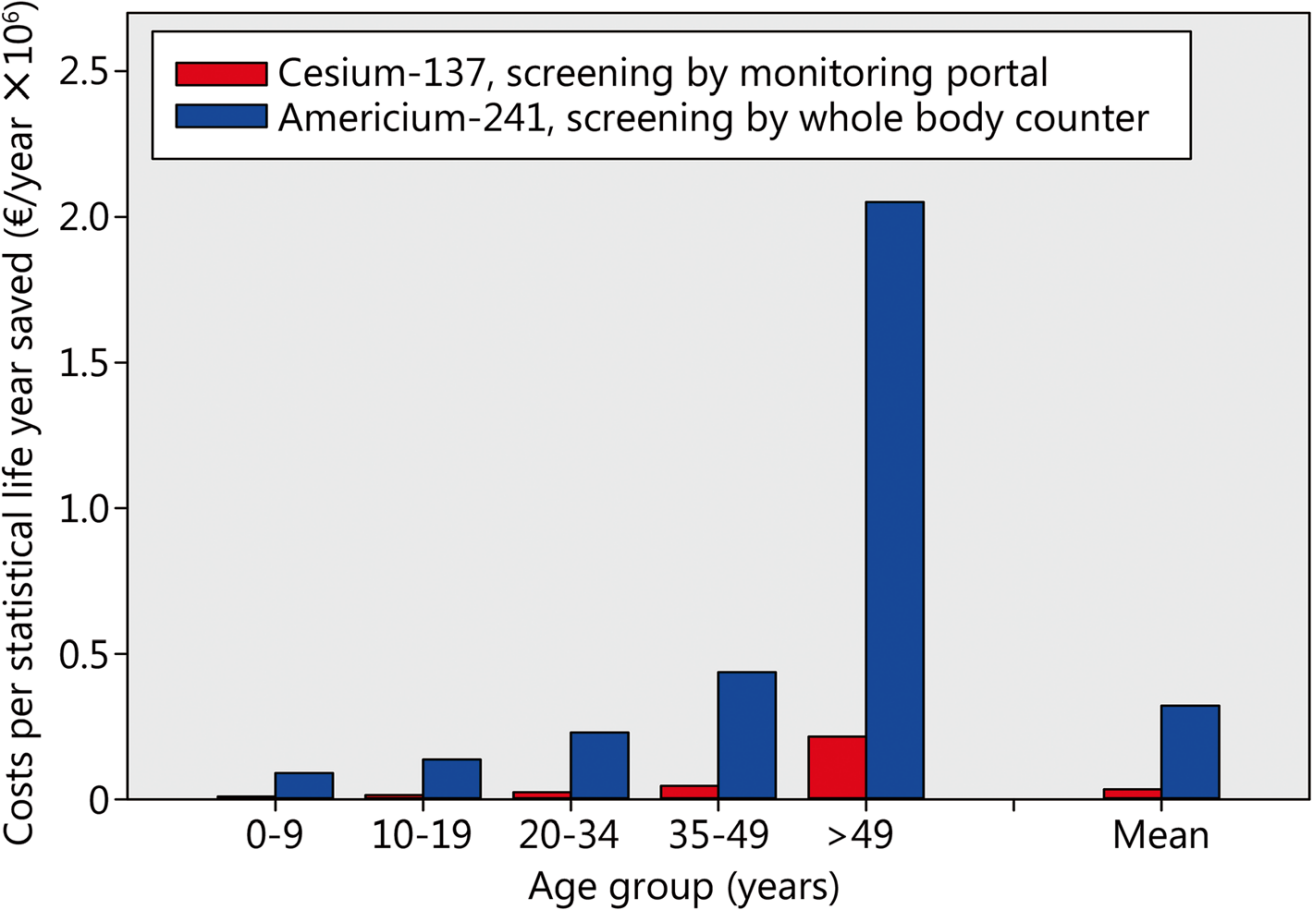
Costs to save a statistical life year in different age groups after the acute inhalative incorporation of cesium-137 or americium-241 if implementing an “urgent approach” treatment strategy with decorporation starting 12 h after radioactivity exposure. Assumptions: Decorporation treatment threshold level 200 mSv; 1% of the potentially contaminated victims actually need treatment; purchase costs of equipment and antidotes: see method section without discounts

Assuming that 1% of the potentially contaminated victims have incorporated cesium-137 amounts leading to a dose of over 200 mSv and thus need treatment, the availability of 6 monitoring portals (113,400 antidote daily doses needed for a treatment of 90 days) is associated with the highest economic efficiency (Fig. 3). The costs of saving 1 statistical life year amount to 33,860 € if considering the average of all age groups (Fig. 4). This situation corresponds to initiating treatment in all victims on the first day (assuming availability of antidotes 12 h after exposure) and thereafter immediately starting screening and completing it in all persons before a second antidote dose would normally be administered. Only patients with confirmed substantial radioactivity incorporation are treated in the further course.

Whole-body counters are not a reasonable alternative for cesium-137 activity measurements from an economic viewpoint. The highest efficiency if relying solely on whole-body counters would be achieved with 25 units (Fig. 3), but the costs to save one statistical life year would amount to 349,312 €, i.e. about 10 times the amount spent if using monitoring portals.

In the case of americium-241, mathematically the highest efficiency would also be reached with 6 monitoring portals and compared to cesium-137 values differ only slightly due to relatively small daily price differences between the two antidotes. However, from a clinical point of view, it is not meaningful to base procurement decisions on relative efficiency values in this case, as the americium-241 activities that can be detected by a monitoring portal are far above the treatment threshold levels usually considered as acceptable (see section 3.1: 922 mSv with reinforced shielding vs. a treatment threshold of 200 mSv) (in Fig. 3 efficiency values for monitoring portals and americium-241 are only shown for completeness). Efficiency must not be considered independently of clinical effectiveness and a satisfactory detection of americium-241 requires the use of a whole-body counter.

In the case of americium-241 and 1% of the victims actually needing treatment, the highest efficiency is also attained with 25 whole-body counters, corresponding to a daily screening capacity of 2500 people/d (Fig. 3). This is much less than the capacity of 60,000/d achieved with 6 monitoring portals and may be associated with organizational issues (e.g. necessity of appointments over many days) or psychological fears related to uncertainties inducing unnecessary workloads in emergency departments at hospitals. The costs for saving 1 year statistical lifetime in case of americium-241 incorporation (322,343 €/year) is in a comparable order of magnitude than in the case of the use of whole-body counters to detect cesium-137 (Fig. 4). This is the consequence of the fact that in both cases a 90-day treatment has a comparable efficacy (53.8% for cesium-137 and 54.2% for americium-241). However, in the case of americium-241 there is no cheaper alternative if a satisfactory detection limit is to be ascertained and so efficiently preparing for a “dirty bomb” attack with americium-241 is more expensive and cost-effectiveness less.

Efficiency values and the costs of a statistical life year strongly depend on the way the available equipment is used. Increasing the screening capacity of every monitoring portal to 2000 people/h seems possible from the detection limit for cesium-137, but might be difficult from a practical point of view. However, it seems quite possible to extend the effective daily working time from 10 to 20 h a day, if the number of technically trained personnel is available. This would increase the daily screening capacity to 20,000 persons/d and unit. Efficiency would be achieved with 3 monitoring portals and costs would be reduced to 29,823 € per statistical life year saved. Extending working hours to 20 h per day would also permit to achieve efficiency with only 15 whole-body counter units (costs: 232,362 € per saved life year). On the other side, realizing a better detection limit for americium-241 by extending individual measurement times to 15 min (i.e. screening capacity of 4 persons/h and 40/d if working 10 h) means that 38 units will be necessary for efficiency, and costs of a statistical life year saved will amount to 501,859 €. Therefore besides equipment, the availability of technically trained staff is a further important determinant of the number of screening units required to reach efficiency.

### Further factors affecting efficiency: the age of the victims, the fraction of the victims needing treatment and the treatment indication threshold level

The sensitivity to radiation depends on age with children and young adults statistically losing more lifetime as elderly people if irradiated (Table 1). The benefit of decorporation treatment is higher in younger age groups and this is reflected in lower costs to save a statistical life year (Fig. 4, Table 1). In the age group over 50 years, the costs amount more than 20 times the costs in children below 10 years of age. The costs to save life time after americium-241 incorporation are about 10 times higher than after cesium-137 exposure, as expected from the more expensive screening equipment in combination with a lower screening capacity.

**Table 1:** Costs to save a statistical life year in different age groups

Age group (years)	Lifetime saved (d/mSv)	Costs (€/year saved)	
		Cesium-137 intake	Americium-241 intake
0–9	1.5	9481	90,259
10–19	0.99	14,366	136,756
20–34	0.59	24,104	229,483
35–49	0.31	45,879	436,736
> 50	0.066	215,450	2,051,100

Assumptions: Acute inhalative incorporation of cesium-137 or americium-241 and implementing an “urgent approach” treatment strategy with decorporation starting 12 h after radioactivity exposure; Decorporation treatment threshold level 200 mSv; 1% of the potentially contaminated victims actually needing treatment; purchase costs of equipment and antidotes: see method section without discounts; Source for the lifetime saved in the age groups: [36]

The efficient resource mix and the costs of a life year saved heavily depend on the proportion of potentially contaminated victims who actually need decorporation treatment. In the case all victims would need decorporation treatment (100%), it does not make sense to keep screening equipment available, as they are costly but do not allow to spare on antidote stockpiling. The lower the percentage of potentially contaminated people that actually need treatment, the more important are high screening capacities to achieve efficiency, in the case of cesium-137 as well as americium-241 incorporation (Table 2). However, the costs to save one statistical life year strongly increases with lower proportions of patients needing treatment and for 0.01% reaches huge amounts of several million € after cesium-137 but particularly americium-241 incorporation (Table 2). This is also intuitively easy to understand, as the investment permits to identify only a few patients needing treatment (0.01% of 60,000 means 6 people) with each of these persons gaining only very short additional lifetime (for an indication threshold level of 200 mSv: 45 days/patient).

**Table 2:** Number of monitoring portals or whole-body counters (N) associated with economic efficiency and corresponding costs of a statistical life year saved depending on the percentage of potentially contaminated victims actually needing decorporation treatment

	Cesium-137 monitoring portal		Americium-241 whole-body counter	
	*N*	€/year	*N*	€/year
0.01%	6	2,183,833	25	31,336,742
0.1%	6	229,312	25	3,141,834
1.0%	6	33,860	25	322,343
10%	6	14,315	20	40,212
25%	6	13,012	20	21,314
50%	3	12,565	15	14,932
75%	3	12,402	10	12,609
90%	2	12,341	5	11,636
100%	0	12,280	0	10,529

Assumptions: Scenario with 60,000 potentially contaminated people; purchasing cost of a monitoring portal 100,000 € and screening capacity of 10,000 people/day and unit; purchasing cost of a whole-body counter 500,000 € and screening capacity of 100 people/day and unit. Antidote requirements depending on screening capacities and percentage of victims needing treatment calculated according to the algorithm described in [23]

**Fig. 5:**
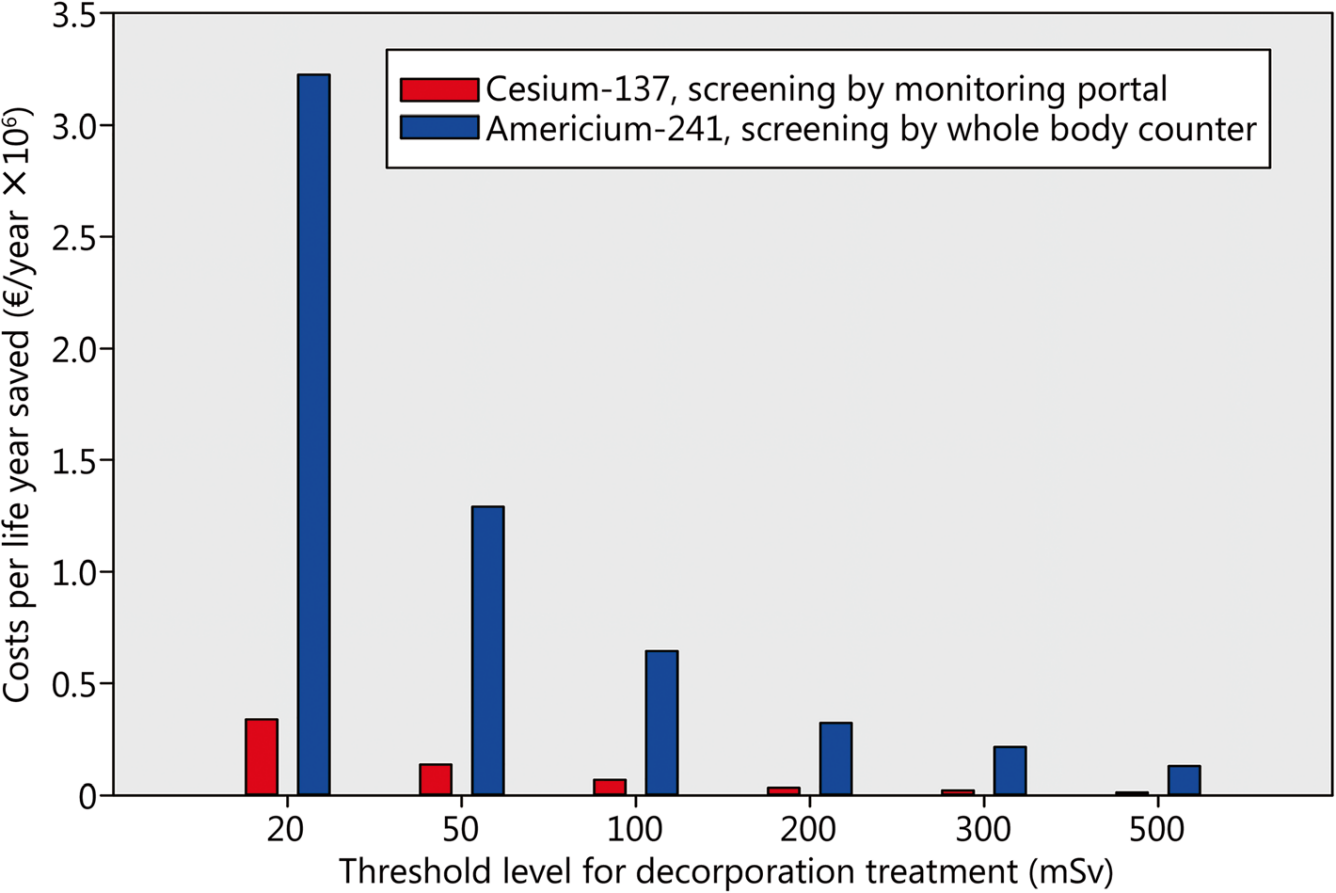
Costs of a statistical life year depending on the threshold level fixed for the indication of decorporation treatment and a resource mix corresponding to optimum efficiency (1.000). Assumptions: Scenario with 60,000 potentially contaminated people and 1% of them actually needing treatment; purchasing cost of a monitoring portal 100,000 € and screening capacity of 10,000 people/day and unit; purchasing cost of a whole-body counter 500,000 € and screening capacity of 100 people/day and unit

The fraction of the victims needing treatment will also depend on the threshold value fixed as a limit to initiate decorporation therapy. The lower the value, the higher the costs of a life year saved (Fig. 5, Table 3). It must however be emphasized that the costs calculated are the consequence of the fact that we consider all victims needing treatment to have absorbed a committed effective dose equal to the threshold. Therefore, the direction the cost-effectiveness varies has to be considered, but the monetary values should be considered just as overestimated indications.

**Table 3:** Costs of a statistical life year depending on the threshold level fixed for the indication of decorporation treatment and a resource mix corresponding to optimum efficiency

Threshold level (mSv)	Cesium-137		Americium-241	
	Total lifetime saved (years)	Costs per life year saved (€/year)	Total lifetime saved (years)	Costs per life year saved (€/year)
20	7.432	338,598	7.483	3,223,431
50	18.58	135,439	18.71	1,289,200
100	37.16	67,720	37.41	644,772
200	74.32	33,860	74.83	322,343
300	111.47	22,575	112.24	214,905
500	185.8	13,544	187.1	128,920

Assumptions: Scenario with 60,000 potentially contaminated people and 1% of them actually needing treatment; purchasing cost of a monitoring portal 100,000 € and screening capacity of 10,000 people/day and unit; purchasing cost of a whole-body counter 500,000 € and screening capacity of 100 people/day and unit

### The impact of discounts on the efficiency of the resource mix

Discounts on antidote purchase prices affect the number of screening equipment units required for efficiency and the costs of statistical lifetime saved (Table 4). In the case of cesium-137, the number of monitoring portals needed for an efficient mix will remain at 6 as long as the discount for Prussian Blue does not exceed 40%. In the case of americium-241, even very small discounts will affect the number of mobile whole-body counters needed: 25 units are needed only as long as discounts for Ca(DTPA) does not exceed 3–4%.

**Table 4:** Number of screening equipment units needed to reach optimum efficiency depending on the discount granted on antidote prices

Discount on antidote prices	Cesium-137		Americium-241	
	Monitoring portal units	Costs per life year saved (€/year)	Whole-body counter units	Costs per life year saved (€/year)
−10%	6	31,281	20	304,674
−30%	6	26,124	20	266,666
−50%	3	20,307	15	224,202
−70%	3	13,799	15	174,612
−90%	2	6620	5	100,042

Assumptions: Scenario with 60,000 potentially contaminated people and 1% of them actually needing treatment; purchasing cost of a monitoring portal 100,000 € and screening capacity of 10,000 people/d and unit; purchasing cost of a whole-body counter 500,000 € and screening capacity of 100 people/d and unit

Similarly, changes in the price of the screening equipment will particularly influence the number of whole-body counters needed to reach efficiency: Whereas 6 monitoring portals are the efficient solution in the price range of ±50% (50,000–150,000 €/unit), the number of whole-body counters to reach an efficient resource mix will vary from 20 (750,000 €/unit) to 30 units (250,000 €/unit) (Table 5).

**Table 5:** Number of screening equipment units needed to reach optimum efficiency depending on the purchase price of the equipment

Monitoring portal			Whole-body counter		
Purchase price (€)	Units for efficiency	Costs per life year saved (€/year)	Purchase price (€)	Units for efficiency	Costs per life year saved (€/year)
50,000	6	29,823	250,000	30	232,362
75,000	6	31,841	375,000	25	280,582
100,000	6	33,860	500,000	25	322,343
125,000	6	35,878	625,000	20	357,088
150,000	6	37,896	750,000	20	390,497

Assumptions: Scenario with 60,000 potentially contaminated people and 1% of them actually needing treatment; no discounts for antidotes

### Resource requirements for a comprehensive preparedness including cesium-137 and americium-241 involvement

The previous results apply to preparedness to attacks involving cesium-137 or americium-241 considered separately. Whole-body counters are necessary for the measurement of americium-241 activity because of their lower detection limits. However, the same whole-body counters may also be used to screen people having incorporated cesium-137. Therefore, the question whether it is justified to invest in the procurement of additional monitoring portals when whole-body counters are available seems justified.

Assuming 1% of victims actually needing decorporation treatment after americium-241 incorporation, efficiency is reached with 25 whole-body counters (assumption for capacity 10 people/h, 100 people/d and unit, 2500 people/d for 25 units, price 500,000 €/unit) corresponding to a total of 12.5 million € purchasing costs. The costs for the required Ca(DTPA) daily doses amount to 11.62 million € (no discounts). With 25 whole-body counters available, the corresponding needed Prussian Blue antidotes will cost 13.46 million €. The whole costs for preparedness relying only on whole-body counters for measurement will amount to 37.58 million € (Table 6).

**Table 6:** Costs related to preparedness based on whole-body counters only (option 1) or a combination of whole-body counters and monitoring portals (option 2)

	Option 1		Option 2	
	Units / daily doses	Costs (million €)	Units / daily doses	Costs (million €)
Whole-body counter	25	12.5	25	12.5
Monitoring portal	0	0	6	0.6
Ca(DTPA)	796,500	11.62	796,500	11.62
Prussian Blue	796,500	13.46	113,400	1.92
Total costs	–	37.58	–	26.64

The number of units of screening equipment corresponds to efficiency for the resource mix if considering preparedness to a dirty bomb attack with americium-241 or cesium-137 separately. The daily screening capacity of 25 whole-body counters is assumed to be 2500 victims/d and for the monitoring portals 60,000 victims/day. Further assumptions: Large-scale scenario with 60,000 potentially contaminated victims and 1% actually needing treatment. For the equipment and antidote prices see the text without discounts. -: Not applicable

The purchase of 6 monitoring portals will amount to 0.6 million €, but considering the much higher screening capacity per hour (100 people/h, 1000 people/d and unit, 60,000 people/d for 6 units), the number of Prussian Blue daily doses required in stock (113,400 instead of 796,500 daily doses) will be much lower with a purchase value even without discounts of 1.9 million €. Therefore the total costs of preparedness including measurement equipment (monitoring portals and whole-body counters) and antidotes (Ca(DTPA) and Prussian Blue) will total 26.64 million € and thus will be roughly 30% less than relying solely on whole-body counters (Table 6).

## Discussion

Our results show that high screening capacities are a major determinant to achieve efficiency in the resource mix when preparing to cope with the medical consequences of a dirty bomb attack. High screening capacities reduce the costs of a statistical life-year saved, but probably there are also indirect cost reducing-effects not reflected in our figures, like fewer visits of “worried well” patients at emergency departments.

Efficiency values and the precise optimum antidote screening capacity combination depend on several factors that in part can be voluntarily fixed by planners or are outside of their influence. The decision on the threshold levels justifying a decorporation treatment is a normative decision as long as scientifically based limits excluding stochastic radiation damages are not known. The committed effective doses used to define thresholds are cumulative doses over 50 years after radionuclide(s) incorporation (or over 70 years for children). However, this dose is not evenly distributed over the whole time period [45]. Based on the assumption that the area under the whole-body activity time curve is proportional to the dose, around 50% of the committed effective dose from acute cesium-137 incorporation is absorbed within 3 to 4 months and about 90% within the first year. This is not surprising as, despite a physical decay half-life of 30 years, the biological half-life is in a range of 70 to 130 days (more precisely, the retention follows a two exponential decay: R(t) = 0.1 × e^(− 0.347 × *t*)^ + 0.9 × e^(− 0.00630 × *t*)^) [43]. This means that a committed effective dose of 200 mSv will lead during the first year after incorporation to an effective dose of much more than 20 mSv which is the dose limit for workers exposed to radiations according to German occupational regulations [46]. Thus, fixing threshold levels for decorporation treatments may be based on considering just the total committed effective dose, i.e. the total lifetime saved on average by the treatment (threshold × efficacy × 0.42 d/mSv) or alternatively it may be based on radiation protection regulations already established for other purposes, in which case the situation is more complex and the threshold will depend on the particular radionuclide, the physicochemical properties of the compound involved and the invasion pathway. Moreover, age may be an additional factor that may be considered when making decisions on threshold levels that may be fixed at a lower level for children as younger people are more sensitive to radiation [31, 47].

The optimum mix of countermeasure resources depends on the prices of the screening equipment and antidotes. Thus, our quantitative results apply only to the present conditions in Germany, although we believe that high screening capacities are probably of value in many other countries. Price levels vary among countries and in particular national regulations on the price settings of pharmaceutical products differ, ranging from free market rules in the US to highly regulated often formula-based mechanisms, e.g. in Japan [48]. However, purchase prices of antidotes by authorities are prone to bargaining and deals. On the other side, it should not be overseen that the market for uncommon equipment and antidotes represent a small market segment and the number of manufacturers may be limited or even monopoly exists. Before deciding on quantitative investments, a thorough market survey should be performed and information from the manufacturers gathered.

Operational costs and in particular the costs of the personnel operating the screening equipment and distributing antidotes or giving advises on the treatments have not been considered in our calculations. The reason is that we feel that it is not justified to keep personnel constantly on alert only to cope with nuclear or radiological emergencies that may happen anytime, but are nevertheless very uncommon. In particular from a psychological point of view this would be a very frustrating occupation. It seems much wiser to organize nuclear and radiological emergency rescue activities in the frame of a task force involving personnel mainly assigned to other but related daily activities (e.g. technicians in laboratory dealing with radiological issues, paramedics) as practiced at the Bundeswehr Institute of Radiobiology [49]. This does not preclude that the additional task to staff teams for emergency situations should be taken into account in the total staffing of the institution. Moreover, staffing must be quantitatively adapted to the screening equipment available and to the effective daily working time targeted in the case of an emergency.

Among the factors that cannot be influenced by emergency planners is the scale of the scenario. Our computations are based on the US National Planning Scenario Nr. 11. Although it is a large-scale scenario, it seems realistic to us when considering possible sites of dirty bomb attacks in German cities with their particular density of population and/or visitors [23].

Another determinant that is extremely difficult to predict but highly relevant for countermeasure efficiency assessments is the fraction of the potentially contaminated victims that actually need decorporation treatment. Estimations vary greatly. Based on the data known from the Goiânia incident a figure of 1% has been suggested for a dirty bomb attack [24]. Although no explosion occurred in this incident, it was reported that a primary cause of area contamination in Goiânia was nevertheless by atmospheric dispersion [50, 51]. For the scenario used in this study, the US Department of Health states that probably 40 to 60% of the contaminated victims will need treatment [37]. The chosen threshold level for decorporation treatment indication will be an important determinant of the fraction of the victims actually needing therapy.

Based on information given on the scenario, we calculated the mean radioactivity concentration in the radioactive plume before complete deposition of the particles depending on their size (100 μm particles, deposition velocity 0.3 m/s; 5 μm particles, 0.002 m/s) [52]. Depending on the duration of stay in the proximity of the detonation point, we derived the activity inhaled (assumed breathing rate 3.3 x 10^− 4^ m^3^/s) and determined the absorbed doses using IMBA software. Assuming that most particles are of large size (100 μm particles 100%) as given in the scenario, the committed effective dose (50 years) by internal contamination would amount to only 4.2 mSv if inhaling the air of the plume for 30 min. In the case of a larger proportion of small size particles (e.g. 100% of 5 μm particles), the dose would amount to 140 mSv or 278 mSv if staying in the plume for 30 min or 60 min, respectively. The values are in a range where the decision to initiate decorporation treatment or not will depend on the selected indication threshold value. Our estimates are based on many assumptions and actually the absolute number and percentage of victims needing treatment will depend on the combination of many factors: The explosive load in the bomb, the construction of the device, the radionuclide(s) and the physico-chemical properties of the compound, the total amount of radioactivity, the location of the detonation and the density and position of people in the surrounding areas, the meteorological conditions and the architecture of buildings and blocks affecting the dispersion of the particulates [12].

An additional question is whether the health benefits achieved are worth the specific investments in the preparedness for a dirty bomb attack. Besides the ethical side, the issue should also be considered from the perspective of health economics, as financial inputs in such specific countermeasures will probably lessen the resources available for other fields of health care. The issue is particularly difficult as the probability of occurrence of a dirty bomb attack cannot be predicted and therefore the concept of risk as a product of probability and damage cannot be used. Risk is just a function of the intention and the will of the terrorists to harm as well as the availability of means and abilities [53].

**Fig. 6:**
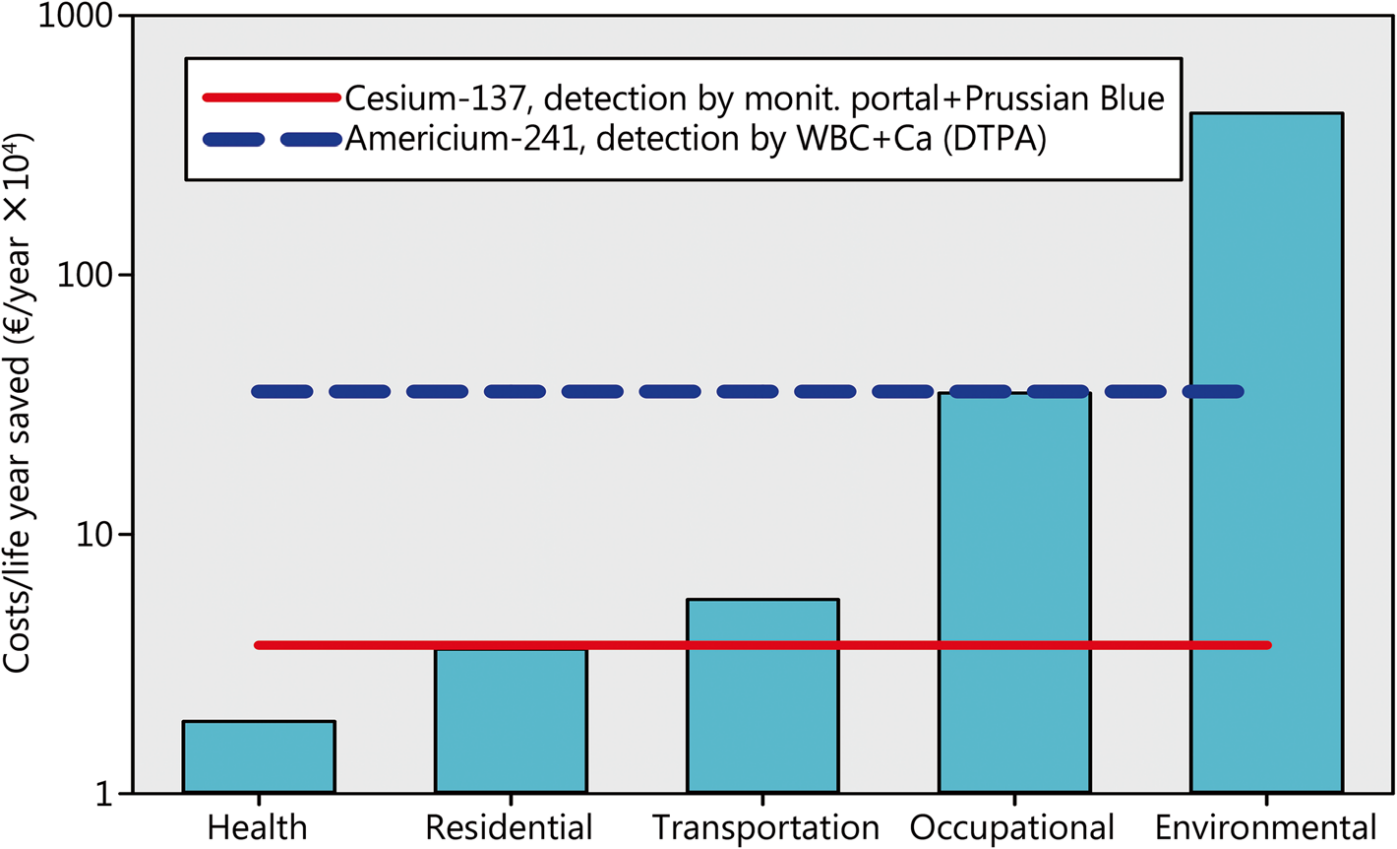
Costs per life year saved in various sectors of society (columns, logarithmic scale) (data from [56]) and values achieved by preparedness for a dirty bomb attack with cesium-137 or americium-241. Exchange rates 1 € = 1.1 US-$; monit. Port.: Monitoring portal; WBC: Whole body counter

Life years saved is a popular metric to assess effectiveness as it accounts for premature deaths at all ages and the comparison of costs incurred to save a statistical life year permit to select among mutiple competing policies [54]. However, there is an enormous range of values for different interventions targeted at reducing health risks. Alone among medical lifesaving procedures, the costs per life year saved have been reported to range from 92.14 US-$ for a cesaerian section or 102.67 US $ for an appendectomy at the lower end to 14,087.50 US-$ for a simultaneous prancreas/kidney transplant and 20,472.11 US-$ for a heart transplant at the upper end [55]. Depending on the methodology, values differ among authors (e.g. heart transplantation for younger patients and favorable prognosis 3600 US-$/year; heart transplantation for patients with terminal heart disease 100,000 US-$/year) [56]. Comparing cost-effectiveness ratios show astonishing differences between sectors of society with increasing costs of interventions to save lifetime in the order health care (median 19,000 US-$/year), residential (36,000 US-$/year), transportation (56,000 US-$/year), occupational (350,00 US-$/year) and environmental safety (4,200,000 US-$/year) [56]. Costs to save lifetime are particularly high for interventions with the goal to control chemical hazards [56]. Putting investments into screening capacities and decorporation antidotes to prepare for a dirty bomb attack in this broader perspective, despite all moderating variables affecting the costs of a life year saved, suggests that cost-effectiveness is less than for health care interventions, but better than for environmental improvements (Fig. 6).

Another approach may be based on the concept of “value of a statistical life” (VSL). A value of statistical life (VSL) is the amount that a group of people is willing to pay (WTP) for fatal risk reduction in the expectation of saving one life [57]. We already used this methodology to assess at the microeconomic level of an individual patient the costs-benefit ratio of cesium-137 decorporation by a Prussian Blue treatment and stockpiling [58]. Based on two different VSL values determined for Germany in wage-risk studies using different methodologies [59] and similarly to Oka [36], applying these values to a middle-aged person with on the average a remaining statistical life expectancy of 40 years, we determined the value of a single life year with 112,275 €/year (4.491 million €/40 years) or 41,150 €/year (1.646 million €/40 years). The latter value seems quite low compared to the bulk of the VSL-literature, but roughly corresponds to the value of a quality-adjusted life year (QALY) used by the National Institute for Health and Care Excellence (NICE) in the United Kingdom when assessing the cost-effectiveness of new technologies (20,000–30,000 £ equivalent to 26,000–39,000 € at market exchange rates) [60, 61]. Assuming 1% of the potentially contaminated victims to actually need treatment, a threshold level for treatment indication of 200 mSv and an efficient (1.000) resource mix, our results indicate that the investment for the preparedness with portal monitors to a dirty bomb attack with cesium-137 is worth the benefit achieved (33,860 €/year saved) compared to spending in medical care, even if applying the standards of NICE. Investments in mobile whole-body counters to an extent to reach economic efficiency to cope with attacks involving americium-241 are far more expensive (322,343 €/year saved), but as already mentioned they are still in a comparable range as interventions in occupational or environmental safety. As explained in the Method section, it should also be remembered that because of our methodology (the lifetime saved is calculated based on the fixed threshold level and no dose above), there is a tendency that in our results the total benefit of decorporation treatment expressed as total life time saved is underestimated and the costs of a life year saved therefore overestimated.

## Conclusion

Based on a concrete dirty bomb attack scenario and estimated price levels for screening equipment and antidotes, it is possible to calculate from an economic point of view the optimum mix of medical countermeasure resources necessary to achieve the best medical results using an “urgent treatment” approach. High screening capacities using monitoring portals or mobile whole-body counters, depending on the radionuclide(s) expected, are of major importance to achieve a high efficiency of the resource mix. Assessing the cost-effectiveness ratio in comparison to policy interventions in other sectors of society is a much more difficult task. Among the determinants of the costs of a saved life year, the fraction of the potentially contaminated victims actually needing treatment is a crucial point and deserves to be grasped more precisely. Whether the costs for the lifetime saved by a high preparedness level for dirty bomb attacks should be compared only to the cost-effectiveness of relatively cheap interventions in medical care or to much more expensive interventions for risk reductions in the occupational or particularly in the environmental sector is a decision of a political nature.

## Data Availability

The data used for the computations in this analysis are freely available in the literature.

## Abbreviations

Ca (DTPA): Calcium (diethylenetriaminepentaacetic acid)
Cs: Cesium
CsCl: Cesium chloride
€: Euro
IMBA: Integrated modules for bioassay analysis
keV: Kiloelectronvolt
mSv: Millisievert
RDD: Radiological dispersal device
US-$: US dollar
VSL: Value of a statistical life
WTP: Willingness to pay

## References

[1] Edsall K, Walter FG (2003). Radiological and nuclear incidents and terrorism. Advanced hazmat life support (AHLS) provider manual.

[2] NCRP (2010). Population monitoring and radionuclide decorporation following a radiological or nuclear accident.

[3] Stricklin D, Millage K, Rodriguez J, McClellan G (2014). Americium-241 decorporation model. Technical report DTRA-TR-15-02.

[4] International Atomic Energy Agency (IAEA) (1988). The radiological accident in Goiânia.

[5] Krock L, Deusser R (2003). Dirty bomb homepage. Chronology of events. Nova Science Programming on Air and Online.

[6] Hall EJ, Giaccia AJ (2006). Radiobiology for the radiologist.

[7] Radiology Key. Radiologic terrorism. https://radiologykey.com/radiologic-terrorism/.Accessed 10 Jan 2020.

[8] Von Winterfeldt D, Rosoff H (2007). A risk and economic analysis of dirty bomb attacks on the ports of Los Angeles and Long Beach. Risk Anal.

[9] Haines JR (2014). “Dirty bomb”: Reason to worry? Explosive radiological dispersal devices and the distribution of dangerous radiological material worldwide.

[10] Connell LW. Dirty bomb risk and impact. A systems analysis overview. Contract DENA0003525 for the US Department of Energy: Sandia National Laboratories; 2017. https://prod-ng.sandia.gov/techlib-noauth/access-control.cgi/2017/179121r.pdf. Accessed 15 Jan 2020.

[11] Harper FT, Musolino SV, Wente WB (2007). Realistic radiological dispersal device hazard boundaries and ramifications for early consequence management decisions. Health Phys.

[12] Andersson KG, Mikkelsen T, Astrup P, Thykier-Nielsen S, Jacobsen LH, Schou-Jensen L (2008). Estimation of health hazards resulting from a radiological terrorist attack in a city. Radiat Prot Dosim.

[13] Rump A, Becker B, Eder S, Lamkowski A, Abend M, Port M (2018). Medical management of victims contaminated with radionuclides after a “dirty bomb” attack. Mil Med Res..

[14] Yan T, Lin G, Wang M, Lamkowski A, Port M, Rump A (2019). Pharmacological treatment of inhalation injury after nuclear or radiological incidents: the German and Chinese approach. Mil Med Res.

[15] Weickhardt U (2001). Der Strahlenunfall. Informationsschrift zur Behandlung von Strahlenverletzten.

[16] Roessler G (2007). Why Po-210?. Health Phys News.

[17] Altagracia-Martinez M, Kravzov-Jinich J, Martínez-Núnez JM, Ríos-Castaneda C, López-Naranjo F (2012). Prussian blue as an antidote for radioactive thallium and cesium poisoning. Orphan Drugs: ResRev.

[18] Melo DR, Lipsztein JL, Leggett R, Bertelli L, Guilmette R (2014). Efficacy of Prussian blue on 137 Cs decorporation therapy. Health Phys.

[19] Rump A, Stricklin D, Lamkowski A, Eder S, Abend M, Port M (2016). Reconsidering current decorporation strategies after incorporation of radionuclides. Health Phys.

[20] Rump A, Stricklin D, Lamkowski A, Eder S, Abend M, Port M (2016). The impact of time on decorporation efficacy after a “dirty bomb” attack studied by simulation. Drug Res..

[21] Rump A, Stricklin D, Lamkowski A, Eder S, Abend M, Port M (2017). The incorporation of radionuclides after wounding by a “dirty bomb”: the impact of time for decorporation efficacy and a model for cases of disseminated fragmentation wounds. Adv Wound Care.

[22] Grappin L, Berard P, Beau P, Carbone L, Castagnet X, Courtay C (2006). Exposition aux actinides. Bilan injection de Ca-DTPA dans les centres CEA-Cogema. Rapport CEA-R-6097.

[23] Rump A, Stricklin D, Lamkowski A, Eder S, Abend M, Port M (2017). Analysis of the antidote requirements and outcomes of different radionuclide decorporation strategies for a scenario of a “dirty bomb” attack. Am J Disaster Med.

[24] Perez M, Carr Z (2007). Development of stockpiles for radiation emergencies. Report of the radio-nuclear working group. WHO consultation meeting on development of stockpiles for radiation and chemical emergencies.

[25] Liste G (2015). Fachinformation Radiogardase®-Cs Kaps.

[26] Liste G (2015). Fachinformation Ditripentat-Heyl®(DTPA).

[27] Homeland Security Council (HSC) (2004). Radiological attack – Radiological dispersal devices, Scenario 11. HSC. Planning scenarios. Executive summaries. Version 2.0.

[28] Youngman MJ (2015). Review of methods to measure internal contamination in an emergency. J Radiol Prot.

[29] Wood R, Sharp C, Gourmelon P, Le Guen B, Stradling GN, Taylor DM (2000). Decorporation treatment – medical overview. Radiat Protect Dosim.

[30] Ménétrier F, Grappin L, Raynaud P, Courtay C, Wood R, Joussineau S (2005). Treatment of accidental intakes of plutonium and americium: guidance notes. Appl Radiat Isot.

[31] Rojas-Palma C, Liland A, Jerstad AN, Etherington G, del Rosario PM, Rahola T (2009). TMT Handbook. Triage, monitoring and treatment of people exposed to ionizing radiation following a malevolent act.

[32] Mirion Technologies (RADOS) GmbH (2017). CheckPoint: Gate ™ FastTrack-Fibre ™ Mobile.

[33] Birchall A, Puncher M, Marsh JW, Davis K, Bailey MR, Jarvis NS (2007). IMBA professional plus: a flexible approach to internal dosimetry. Radiat Prot Dosim.

[34] Autorité de Sureté Nucléaire (ASN) (2008). Guide national. Intervention médicale en cas d’évènement nucléaire ou radiologique. Version V 3.6.

[35] Premier Ministre. Secrétariat général de la défense et de la sécurité nationale. Circulaire relative à doctrine nationale d'emploi des moyens de secours et de soins face à une action terroriste mettant en œuvre des matières radioactives. http://circulaire.legifrance.gouv.fr/pdf/2011/03/cir_32735.pdf. Accessed 15 Nov 2019.

[36] Oka T (2012). Application of cost-benefit analysis to the regulation of foodstuffs contaminated with radioactive substances. Jpn J Health Phys.

[37] U.S. Department of Health and Human Services. Public health emergency. national planning scenario # 11. Washington DC; 2015. https://www.phe.gov/Preparedness/planning/playbooks/rdd/Pages/scenario.aspx. Accessed 10 Jan 2020.

[38] Coelli TJ, Prasada Rao DS, O'Donnel CJ, Battese GE (2005). An introduction to efficiency and productivity analysis. 2nd.

[39] Besstremyannaya G (2013). The impact of Japanese hospital financing reform on hospital efficiency: a difference-indifference approach. Jpn Econ Rev.

[40] Asandului L, Roman M, Fatulescu P (2014). The efficiency of healthcare systems in Europe: a data envelopment analysis approach. Procedia Econ Financ.

[41] Rump A, Schöffski O (2018). Pregnancy care in Germany, France and Japan: an international comparison of quality and efficiency using structural equation modelling and data envelopment analysis. Public Health.

[42] Stepan A, Fischer E (2009). Betriebswirtschaftliche Optimierung.

[43] Rääf CL, Falk R, Lauridsen B, Rahola T, Skuterud L, Soogard-Hansen S (2006). Human metabolism of caesium.

[44] Youngman MJ (2017). Transportable system for monitoring internal radioactive contamination in people. Public Health England.

[45] Rump A, Eder S, Lamkowski A, Hermann C, Abend M, Port M (2019). A quantitative comparison of the chemo- and radiotoxicity of uranium at different enrichment grades. Toxicology Lett.

[46] Bundesamt für Justiz (2018). Verordnung zum Schutz vor der schädlichen Wirkung ionisierender Strahlung.

[47] Rodrigues M, Chaput J, Bellman C, Cousins T (2010). Children as vulnerable populations in radiological/nuclear events: discussion scenarios. Radiat Prot Dosim.

[48] Organization for Economic Co-operation and Development (OECD) (2008). Pharmaceutical pricing policies in a global market.

[49] Haupt J, Kaatsch HL, Eder SF, Port M, Rump A (2019). Medizinische Strahlenschutzaktivitäten des Instituts für Radiobiologie der Bundeswehr. Strahlenschutz Praxis.

[50] Da Silva CJ, Delgado JU, Luiz MTB, Cunha PG, De Barros PD (1991). Considerations related to the decontamination of houses in Goiânia: limitations and implications. Health Phys.

[51] Andersson KG, Mikkelsen T, Astrup P, Thykier-Nielsen S, Jacobsen LH, Hoe SC (2009). Requirements for estimation of doses from contaminants dispersed by a ‘dirty bomb’ explosion in an urban area. J Environ Radioact.

[52] Giardina M, Buffa P (2018). A new approach for modeling dry deposition velocity of particles. Atmospheric Environm.

[53] Freudenberg D (2011). Risikoanalyseansätze, Simulation und irreguläre Kräfte. Mil Power Revue der Schweizer Armee.

[54] Graham JD, Johansson PO, Nakanishi J, McDaniels T, Small MJ (2003). The role of efficiency in risk management. Risk analysis and society: an interdisciplinary characterization of the field.

[55] Reisman AM, Farrell K, Leitman M (2018). Value analysis of the costliest elective lifesaving procedures at an academic medical center. J Sci Innov Med.

[56] Tengs TO, Adams ME, Pliskin JS, Safran DG, Siegel JE, Weinstein MC (1995). Five-hundred life-saving interventions and their cost-effectiveness. Risk Anal.

[57] Miller TR (2000). Variations between countries in values of statistical life. J Transp Econ Policy.

[58] Rump A, Stricklin D, Lamkowski A, Eder S, Port M (2018). Benefit-cost analysis of radiocesium decorporation by a Prussian blue treatment and stockpiling. Drug Res.

[59] Spengler H (2004). Kompensatorische Lohndifferenziale und der Wert eines statistischen Lebens in Deutschland. Zeitschrift für ArbeitsmarktForschung (ZAF).

[60] Claxton K, Martin S, Soares M, Rice N, Spackman E, Hinde S, et al. Methods for the estimation of the NICE cost effectiveness threshold. Revised report following referees comments: Centre for Health Economics, Department of Economics and Related Studies. North Yorkshire: University of York, Office of Health Economics, Imperial College London; 2013.

[61] Meacock R, Doran T, Sutton M (2015). What are the costs and benefits of providing comprehensive seven-day services for emergency hospital admissions?. Health Econ.

